# Successful treatment for acute aortic dissection in pregnancy---bentall procedure concomitant with cesarean section

**DOI:** 10.1186/1749-8090-6-139

**Published:** 2011-10-15

**Authors:** Changfa Guo, Demin Xu, Chunsheng Wang

**Affiliations:** 1 Department of Cardiac Surgery, Zhongshan Hospital, Fudan University, Shanghai, China

## Abstract

Acute aortic type A dissection is a life-threatening disease that requires immediate surgical intervention. When dissection occurs during pregnancy, it is of high risk for both the mother and the fetus. In this study, we reported two cases of acute aortic dissection in late pregnancy at 28 weeks and 32 weeks of gestation respectively. After the two patients underwent a cesarean section and delivered a baby, we performed composite graft replacement of the aortic valve, aortic root and ascending aorta, with re-implantation of the coronary arteries into the graft (Bentall procedure) instead of repairing the arch with deep hypothermia and circulation arrest. Both mothers and children survived and recovered well.

## Background

Acute aortic type A dissection in pregnancy is rare, however, of high risk for both the mother and the fetus. Concomitant diseases of Marfan and bicuspid aortic valve disease (BAVD) are of special interest. From Jan 2001 to April 2008, we had two cases of acute aortic dissection in pregnancy (one BAVD patient, and the other Marfan patient), 1.38% (2/145) in all acute aortic type A dissection cases.

## Case report

**Figure 1:**
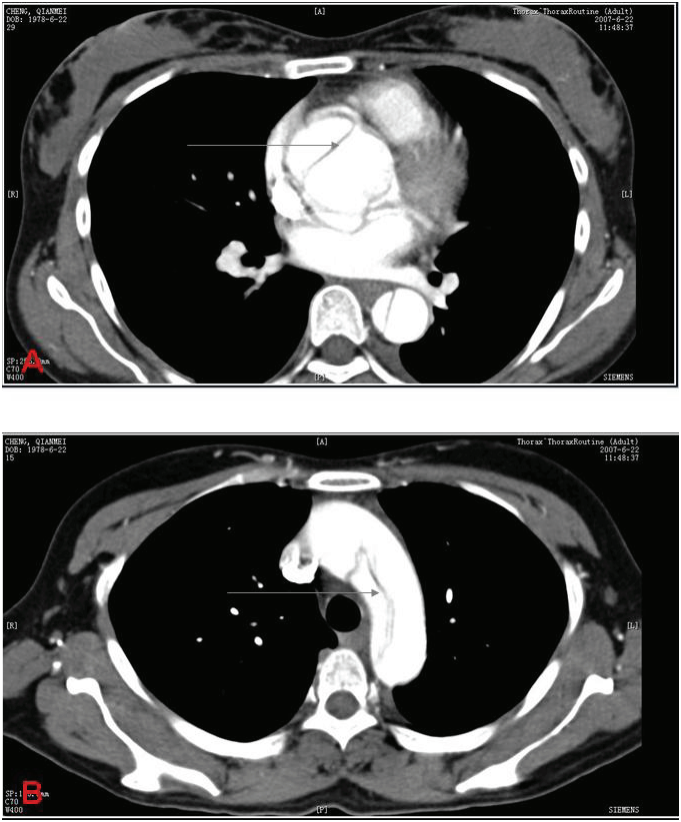
**Aortic dissection in case I with Marfan syndrome**. (A) CT scan: Ascending aortic dissection (grey arrow), (B) CT scan: Aortic arch dissection (grey arrow).

Case I. A 33-year-old Marfan woman, who had had a normal previous pregnancy, was referred because of the acute chest pain with back irradiation. She was at the 28 weeks' pregnancy this time. Echocardiography revealed moderate-severe aortic regurgitation, a dilated aortic root measuring 5.2 cm in diameter, and an acute aortic dissection involving the ascending aorta and the aortic arch, which was also verified by a computed tomography arteriogram (Figure 1-A, B).

**Figure 2:**
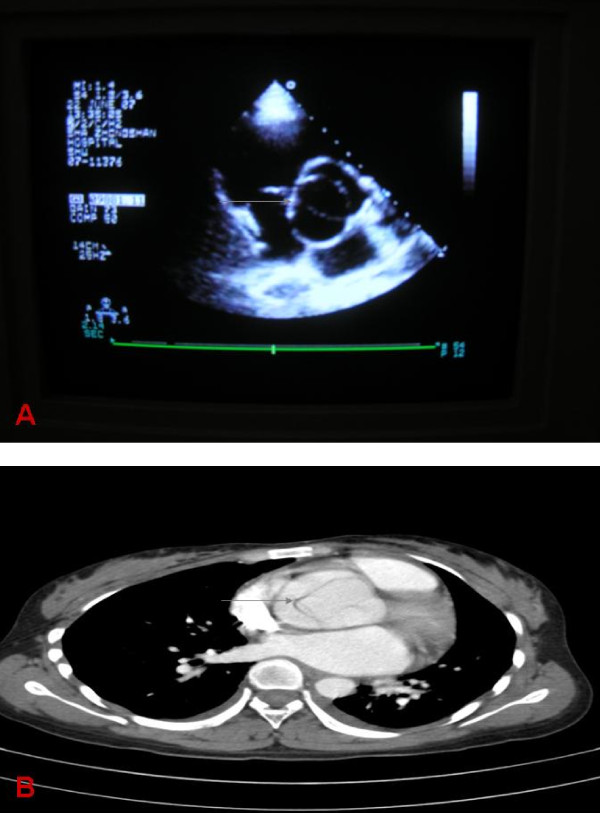
**Aortic dissection in case II with bicuspid aortic valve**. (A) Echocardiography: Bicuspid aortic valve (grey arrow). (B) CT scan: Ascending aortic dissection (grey arrow).

Case II. A 30-year-old woman at 32 weeks of gestation was admitted because of the acute onset of severe chest pain and dyspnea. Her chest x-ray revealed an enlarged heart shadow and widening of the mediastinum. Echocardiography revealed BAVD (Figure 2-A) with moderate aortic stenosis and regurgitation, a dilated aortic root measuring 4.6 cm in diameter, and an acute aortic dissection involving the ascending aorta, which was also verified by a computed tomography arteriogram (Figure 2-B).

These two patients had a negative history of vascular events, high blood pressure, and previous vascular surgery. Under general anesthesia, the cardiopulmonary bypass was built by draining from right atrium and perfusion into the femoral artery or axillary artery. Cesarean section was performed immediately followed by Bentall procedure. After 24 hours of a regular intensive care unit (ICU) postoperative period, the patients were transferred to the ward and then discharged on the 7th postoperative day.

## Discussion

Aortic dissection is rare in young woman, but when it does occur, it is often associated with pregnancy of highest incidence in the third trimester. This is the period of maximal hyper-hemodynamic stress during pregnancy. During this period, there are maximal increases in heart rate, stroke volume, cardiac output, and in left ventricular wall mass and end-diastolic dimension. In addition, estrogen reportedly inhibits collagen and elastin deposition in the aorta, while progestogen accelerates deposition of noncollagen proteins in the aortas. These hormonal effects led to a fragmentation of the reticulin fibers, diminished the amount of acid mucopolysaccharides, and favored loss of the normal corrugation of the elastic fibers [1]. Since these modifications occur in every pregnancy, it is assumed that aortic dissection may have some etiologic factors, such as an inherent defect in the arterial wall. From the literature, Marfan syndrome is responsible for up to 50% of the reports of aortic dissection with pregnancy [2]. Patients with Marfan syndrome have a mutation within the fibrillin gene, and the connective tissue disorder often affects cardiovascular system, and may favor aortic root dilatation and aortic dissection. Abnormal biophysical properties of the aorta in patients with Marfan syndrome were found in normal-sized aortas in patients with magnetic resonance flow mapping [3]. BAVD is another important predisposing factor for aortic dissection. Abnormal elastic properties, similar to the findings in patients with Marfan syndrome, have been demonstrated [4].

Now, the aortic root diameter above which pregnancy is associated with a considerable risk for the occurrence of aortic dissection is still a matter of debate. In a prospective study of 21 Marfan patients, two out of four women with aortic root diameter of between 40 and 43 mm at pregnancy developed an aortic complication [5]. In a retrospective study in 36 patients, four Marfan women with aortic root diameters of between 40 and 45 mm developed aortic dissection [6]. More recently, in 2003, Immer et al. showed that the aortic root enlargement (> 4 cm) during pregnancy in patients with BAVD and Marfan syndrome was associated with a considerable disk for the occurrence of Type A dissection. Not only was the absolute diameter of the aortic root important, but also the rapidity of aortic dilatation was important, and should be taken into account [7]. On the other hand, a recent prospective study of 127 Marfan patients reported by Meijboom and his colleagues demonstrated that pregnancy in women with Marfan syndrome seemed to be relatively safe up to an aortic root diameter of 45 mm [8]. In our cases, the aortic root diameters of the two patients are 46 mm and 52 mm. It seems consistent with Meijboom's study. However, since pregnant women did have aortic dissection with a diameter less than 45 mm, as suggested by previous studies [5-7], we recommend monitor all pregnant women with Marfan syndrome or BAVD very carefully and closely.

Pregnancy and aortic dissection is of no standardized therapeutic concept. In the third trimester of the gestation, especially after 30-weeks of gestation, immediate cesarean section followed directly by cardiac surgery seems to be the most promising option to save the live of the mother and her child. No serious complications have been reported in the literature by adapting this modality of treatment. In our opinion, minimizing surgical repair of acute type A dissection in pregnant patients using Bentall procedure instead of repairing the arch with deep hypothermia and circulation arrest will lead to favorable results at a lower risk. In a recent analysis of 1558 patients with Acute Aortic Type A Dissection, hypothermic circulatory arrest alone resulted in a 30-day mortality rate of 19.4% and a mortality-corrected permanent neurological dysfunction rate of 11.5% [9]. Hence, avoiding the use of this technique and minimizing surgical repair might be important to save the life in our two cases. In case I, though the acute aortic dissection involved the aortic arch, the primary intimal tear was in the ascending aorta, and there was no aneurysm formation in the aortic arch or the distal aorta, and no occlusion of the brachiocephalic artery. So we only did the Bentall procedure without performing the aortic replacement. This patient recovered well at hospital and suffered no complications during 1-year follow-up. In case II, since the acute aortic dissection involved only the ascending aorta, we did the Bentall procedure and cesarean section as well. Both mother and the baby survived and recovered well.

## Consent

Written informed consent was obtained from the next of the kin of the patient involved for publication of this case report and any accompanying images. A copy of the written consent is available for review by the Editor-in-Chief of this journal

## Competing interests

The authors declare that they have no competing interests.

## Authors' contributions

CSW did the operations, conceived of the study, and helped in revising the manuscript critically. CFG, and DMX participated in its design and coordination, and drafted the manuscript. All authors read and approved the final manuscript.
